# Estimating the Impact of Randomised Control Trial Results on Clinical Practice: Results from a Survey and Modelling Study of Androgen Deprivation Therapy plus Radiotherapy for Locally Advanced Prostate Cancer

**DOI:** 10.1016/j.euf.2015.11.004

**Published:** 2016-08

**Authors:** Annabelle South, Wendy R. Parulekar, Matthew R. Sydes, Bingshu E. Chen, Mahesh K. Parmar, Noel Clarke, Padraig Warde, Malcolm Mason

**Affiliations:** a Medical Research Council Clinical Trials Unit, University College London, London, UK; b NCIC Clinical Trials Group, Queen's University, Kingston, ON, Canada; c Department of Urology, The Christie NHS Foundation Trust, Manchester, UK; d Princess Margaret Hospital, Toronto, ON, Canada; e Cardiff University School of Medicine, Cardiff, UK

**Keywords:** Androgen deprivation therapy, Clinical practice, External beam radiotherapy, Impact, Knowledge translation, Prostate cancer

## Abstract

**Background:**

Recent trials have shown that the addition of external beam radiotherapy (EBRT) to androgen deprivation therapy (ADT) improves survival among men with locally advanced prostate cancer.

**Objective:**

To examine the potential impact of these trials on changes in clinical practice and life-years saved.

**Design, setting, and participants:**

A model was developed to examine the impact of changes in clinical practice in the UK. A survey of clinicians who treat men with prostate cancer in the UK and Canada was performed.

**Measurements:**

Outcomes of interest were the proportion of patients treated with different approaches and the predicted number of life-years saved due to changes in clinical practice. Survey data were cross-tabulated and Pearson's χ^2^ tests were applied.

**Results and limitations:**

The survey was completed by 193 clinicians (105 from the UK, 80 from Canada), of whom 70% were clinical/radiation oncologists, 8% were medical oncologists, and 15% were urologists. UK respondents were more likely to report a change in practice in response to the results (44% UK vs 21% Canada). Canadians were more likely to have already been using ADT plus radiotherapy (77% Canada vs 56% UK). The increase in the proportion of patients in the UK treated with ADT + EBRT could result in around 3730–5177 extra life-years at 15 yr from a cohort of 7930 men diagnosed in a single calendar year, compared to if all had been treated with ADT alone.

**Conclusions:**

Trial findings have changed clinical practice, meaning that men with locally advanced prostate cancer are likely to survive longer.

**Patient summary:**

Doctors in the UK have changed practice in response to evidence on the superiority of hormone therapy plus radiotherapy to hormone therapy alone. These changes will improve the survival of men with locally advanced prostate cancer. Further reductions in the use of hormone therapy alone could further improve survival.

## Take Home Message

Clinicians in the UK have changed practice in response to evidence about the superiority of androgen deprivation therapy (ADT) plus radiotherapy to ADT alone. These changes will improve the survival of men with locally advanced prostate cancer. Further reductions in use of ADT alone could further improve survival.

## 1 Introduction

A number of different approaches are used as primary therapy for men with locally advanced prostate cancer, including external beam radiotherapy (EBRT) alone, androgen deprivation therapy (ADT) alone, ADT + EBRT, radical prostatectomy (RP), high–dose-rate (HDR) brachytherapy, and observation with symptomatic interventions when required. There are varying levels of evidence for these approaches.

During the 1980s, Radiation Therapy Oncology Group (RTOG) and European Organisation for Research and Treatment of Cancer (EORTC) trials were carried out comparing EBRT alone to EBRT plus ADT. The results of these trials showed that the EBRT + ADT combination had better outcomes than EBRT alone [1], [2], [3]. However, these studies lacked an ADT-alone arm, and it was therefore not possible to distinguish between benefits arising from ADT or EBRT. Three randomised controlled trials (RCTs) addressing this question have now been carried out and have shown that addition of EBRT to ADT improves overall survival for men with locally advanced prostate cancer [4], [5], [6], [7]. ADT + EBRT is the only therapeutic approach backed by published level 1 evidence for this group of patients. There is evidence from case series and cohort studies to suggest that outcomes after surgery for locally advanced disease can be excellent [8], but there are no randomised trials in this setting.

We examine how evidence showing the benefit of ADT + EBRT has influenced treatment for men with locally advanced prostate cancer in the UK and Canada. We report the results of a model developed to estimate the potential number of life years saved due to changes in practice in the UK. Model inputs were derived from a survey of clinicians. This model may help to quantify potential survival gains at a population level on implementation of clinical trial results.

## 2 Materials and methods

### 2.1 Impact modelling

Overall survival for patients treated with either ADT alone or ADT + EBRT was estimated at yearly time points after randomisation from trial data (PR.3/PR07 [7], trial registration numbers ISRCTN24991896 and NCT00002633) to a maximum of 11 yr, after which fewer than 100 patients were still at risk. These estimates were smoothed and extrapolated for up to 15 yr from randomisation using fractional polynomial models with either two or four degrees of freedom [9]. Models with four degrees of freedom are reported because of their superior fit (*p* < 0.0001). The fitted survival functions are given by

$$\text{ADT}\;\text{alone}\;\text{at}\;\text{time}\;t=\exp\left(-\exp\left(0.482-5.288\times {\left(\text{time}\right)}^{-0.5}+0.0918\times \left(\text{time}\right)\right)\right)$$

and

$$\text{ADT}\;+\;\text{EBRT}\;\text{at}\;\text{time}\;t=\exp\left(-\exp\left(-3.879-1.350\times {\left(\text{time}\right)}^{-1}+1.087\times {\left(\text{time}\right)}^{0.5}\right)\right)\text{.}$$

We estimated the number of extra life years saved due to the shift from ADT alone to ADT + EBRT for various treatment pattern scenarios, each consistent with the survey results, for a cohort of men newly diagnosed with locally advanced disease in the UK in a single year. These were compared to the baseline scenario (73% of men treated using a nonradical approach in 2006–2008, which we assumed to be ADT alone for the model [10]) by calculating the area between the estimated survival curves over 10 and 15 yr. The model was constructed in Microsoft Excel 2010.

### 2.2 Data collection

#### 2.2.1 Survey

To define the input parameters for the model described in Section 2.1, a short online questionnaire was implemented using the Bristol Online Surveys system (www.survey.bris.ac.uk). This collected information on which treatment approaches respondents used for men with locally advanced prostate cancer; awareness of the results of the PR.3/PR07 (NCT00002633) and SPCG-7/SFUO-3 trials (ISRCTN01534787); whether these results had changed their clinical practice; views on the strength of evidence for ADT + EBRT; further evidence needed; barriers to implementation; and priority research questions (Appendix A). Most questions were multiple-choice.

Between November 2012 and June 2013 (more than 1 yr after the PR.3/PR07 results were published) a link to the questionnaire was distributed by e-mail to urologists and oncologists involved in current prostate cancer trials run by the MRC Clinical Trials Unit or the NCIC Clinical Trials Group (primarily UK and Canadian clinicians). Recipients were asked to forward the e-mail to colleagues. It was also distributed to the chairs of the Cancer Network prostate cancer site-specific groups, who were asked to distribute it to the members of their groups. A link to the survey was also tweeted by @MRCCTU and the European Association of Urologists (@uroweb).

Data from the survey were analysed using Stata v.12 (www.stata.com). Responses were cross-tabulated by country and speciality. Pearson's χ^2^ tests were performed to investigate any differences. There was no target sample size.

#### 2.2.2 Other sources

Data for the proportion of locally advanced disease among UK men diagnosed with prostate cancer were taken from the Cancer Research UK website (based on National Cancer Intelligence Network data) [11]. In 2011, 41 736 new cases of prostate cancer were diagnosed in the UK [12]. We estimate that 7930 (19%) involved locally advanced disease, The reference scenario for the proportion of men treated with ADT alone before results for PR.3/PR07 were published was based on data from the National Prostate Cancer Audit of England and Wales [10].

## 3 Results

Table 1 shows the speciality of survey respondents by country. Using data from Table 2 (discussed further below), we generated five plausible scenarios to represent treatment patterns in the UK in 2012–2013 (Table 3).

### 3.1 Modelling results

Table 3 shows an estimate of the number of life years saved by 10 and 15 yr after diagnosis given the new treatment patterns (scenarios 1–5) in comparison to the 2006–2008 treatment pattern had this continued (73% ADT alone) due to the shift from ADT alone to ADT + EBRT; this necessarily ignores other treatment approaches. The estimated cohort comprises 7930 men diagnosed with locally advanced disease per year. If ADT + EBRT use were to increase from 17% to 52% and ADT alone to decrease accordingly (scenario 1), the cohort of men would have 2021 extra life years by 10 yr after diagnosis, and 3730 by 15 yr. If ADT + EBRT use were to increase from 17% to 62% (scenario 2), 2227 and 4111 life years would be saved by 10 and 15 yr, respectively. If ADT + EBRT use were to increase from 17% to 92% (scenario 5), 2804 and 5177 life years would be saved by 10 and 15 yr, respectively.

### 3.2 Survey results

As Table 1 shows, 185 clinicians responded from the countries that recruited to the PR.3/PR07 trial and that were targeted for the survey. Of UK respondents, 89 were from England, 10 from Scotland, five from Wales, and one from Northern Ireland. A further eight responses were from Australia, Germany, Kuwait, the Netherlands, and the USA, presumably reflecting forwarding of the invitation. Their responses are included in the tables, but excluded from the comparative analyses by country. Respondents who reported “other” as their discipline included eight nurses (research nurse, clinical nurse specialist, nurse consultant, or nurse), two research radiographers, one pathologist, one radiation therapist, and one study coordinator. As we do not know how many people received the survey invitation via the different distribution channels (including forwards and tweets), we are unable to assess the response rate.

Table 2 shows the prevalence of treatment approaches by country. The most commonly reported approach for treatment of locally advanced prostate cancer was ADT + EBRT. The next most common approach was ADT alone. UK respondents were more likely to report treating a higher proportion of their patients with ADT alone (*p* = 0.002), and a lower proportion of patients with EBRT alone (*p* = 0.001) than Canadian respondents. Canadian respondents were more likely to report using ADT + EBRT for >90% of patients than respondents from the UK (*p* < 0.041). There were also differences in treatment approaches by discipline. Urologists were more likely to report that a higher proportion of patients in their care were treated with RP than other disciplines (*p* < 0.001), while clinical/radiation and medical oncologists were more likely to report that a higher proportion of patients in their care were treated with ADT + EBRT (*p* < 0.001).

Table 4 shows the survey responses for awareness of the trial evidence, impact on practice, and views on the strength of the evidence. Nearly all respondents were aware of the results of PR.3/PR07. The majority were also aware of the SPCG-7/SFUO-3 results. Clinical/radiation oncologists were more likely to report having read these papers than urologists.

One third of respondents reported that the results had changed their practice, and they now generally use ADT + EBRT, while two thirds reported that it had not changed their practice as they were already generally using ADT + EBRT before the results were known. UK respondents were more likely to report having changed practice in response to the trial results than respondents from Canada, while Canadians were more likely to have already been using this approach (*p* = 0.005).

Most respondents thought that the evidence is sufficiently strong for ADT + EBRT to be the standard of care for men with locally advanced prostate cancer. This varied by speciality: 128/133 (96%) clinical/radiation oncologists, 9/14 (64%) medical oncologists and 20/29 (69%) urologists (*p* < 0.001).

Table 5 presents barriers to implementation of ADT + EBRT. The potential barriers reported as moderate or major by >10% of respondents were toxicity concerns and the attitudes of urology colleagues. UK respondents were less likely than Canadian respondents to report that the attitudes of urology colleagues (*p* = 0.016) or toxicity concerns (*p* < 0.031) were a moderate or major barrier.

Respondents reported that pressing questions for future research are the optimal field for EBRT, the role of RP, the optimal ADT duration, addition of agents to ADT, the use of HDR brachytherapy, and the optimal EBRT dose.

## 4 Discussion

The results of trials comparing ADT alone to ADT + EBRT are well known to clinicians involved in treating men with prostate cancer in Canada and the UK. This is further underpinned by the incorporation of these results into UK, European, and North American guidelines [13], [14], [15]. Previous surveys have shown that prostate cancer guideline recommendations can lead to changes in clinical practice, such as with the National Institute for Health and Clinical Excellence in the UK [16].

In the UK there has been a shift in treatment patterns away from ADT alone towards ADT + EBRT. The evidence suggests that this change in practice will substantially prolong survival (as was observed in British Columbia due to the move away from EBRT with short-term ADT to EBRT with long-term ADT from the late 1990s [17]). However, between 7% and 26% of men with locally advanced prostate cancer were still being treated with ADT alone, which has been shown to be inferior to ADT + EBRT. Some of these men will be unsuitable for EBRT, but there are likely to be others who could benefit yet are not receiving EBRT.

In Canada there has been less of a shift in treatment patterns following the recent results. This is because use of ADT + EBRT was already much higher than in the UK. Canadian practice changed in response to the earlier studies showing the benefit of adding ADT to EBRT, and urologists may have been more willing to refer their patients for radiotherapy than was the case in the UK. At the time when the studies of ADT + EBRT were published, many clinicians in the UK were participating in the ongoing STAMPEDE trial, which subsequently mandated the use of EBRT for locally advanced disease [18] once the results of PR.3/PR07 were published. This, too, may have influenced the change in practice.

Our model illustrates the number of extra life years potentially gained if ADT monotherapy decreases. We hope our findings will encourage teams who routinely use ADT alone for their patients with locally advanced prostate cancer to reflect on their current practice patterns. We also hope that patient groups will find the model results useful in understanding the impact that clinical trials can have on outcomes for the wider patient community, beyond trial participants.

Our model compared treatment scenarios for the UK using survey data with a baseline of 73% of patients in 2006–2008 receiving nonradical treatment (assumed to be ADT alone) according to a national audit of data from England and Wales. In settings where even more men are treated with ADT alone, the gains from a shift to EBRT plus ADT would be greater.

The main barriers reported in our survey to further adoption of ADT + EBRT were concerns about toxicity and the attitude of urology colleagues. In-depth qualitative research may help in understanding how to address these issues.

There are several important limitations to our study. First, the survey was of modest size, with 193 respondents. As clinicians were asked to forward the survey to colleagues, we are unable to assess the response rate and do not know how representative respondents are of the broader clinical community treating men with prostate cancer in the UK and Canada. In particular, we had disappointingly few responses from urologists, despite distribution to the network of investigators involved in the PATCH, PR07, PROMIS, RADICALS, and STAMPEDE trials, and those involved in studies with the NCIC Clinical Trials Group in Canada. Details of the study were also distributed to the chairs of the Cancer Network prostate cancer site-specific groups, who were asked to distribute it to the members of their groups. It is recognised that there has been an increasing trend among urologic surgeons internationally to consider RP as a primary treatment for high-risk locally advanced disease and we are unable to comment accurately about the effect this might have on the results presented here. These deficiencies in our study may impact the generalisability of our findings, and could be an important source of bias if urologists as a group continue to favour surgery or ADT alone with or without supplementary therapies. Our range of scenarios presented reflects the degree of uncertainty.

There is some evidence to suggest that outcomes after RP for locally advanced disease may also be excellent, particularly for younger, fitter men [8], but no RCTs comparing outcomes for RP and ADT + EBRT for locally advanced disease have been reported. There is a lack of directly comparable overall survival estimates from RP, matched for stage and age, in men with locally advanced prostate cancer. Participants in the survey identified the role of RP as a high-priority research question for men with locally advanced prostate cancer. In the absence of RCT data comparing RP versus ADT + EBRT, our model was restricted to looking at the change from ADT alone to ADT + EBRT, ignoring other shifts in treatment approach. We have also extrapolated survival beyond the time periods reported by trials, which may introduce errors into the model.

One further limitation is that the model looks only at survival and does not consider other outcomes that may be important to patients, such as quality of life. While the factors addressed in patient-reported outcomes are important, the lack of comparability between trials in how these are measured makes it particularly difficult to blend such information into the model.

Finally, our model assumes that survival from the treatment approaches will not improve further over the next 10 yr. This is unrealistic; improvements from ongoing research, notably in the castrate-refractory setting, may yet lead to better survival than that predicted by the model.

The best way to measure changes in clinical practice would be through the collection of high-quality routine data from national registries. These should include information on staging and treatments. However, at the time of the baseline estimate for the model, such high-quality, detailed data were not being collected nationally in the UK [19], where the survey results indicated the most potential for impact. For example, there is considerable variation in estimates of what proportion of new prostate cancer diagnoses in the UK involved locally advanced disease. Data reported in the National Prostate Cancer Audit in England and Wales first report indicates that for 2006–2008, 61% of new diagnoses involved locally advanced disease (varying from 42% to 86%, and with completeness of staging information varying from 20% to 78% between cancer networks) [10]. This contrasts markedly with the data from the Eastern Office of the National Cancer Registration Service for 2008–2010 (acknowledged as having the best prostate cancer staging data of the English cancer registries), where 14.8% of new diagnoses were locally advanced disease (personal communication, D. Greenberg, Cambridge, UK). For our model we chose to estimate the cohort size from the value published on the Cancer Research UK website, which is based on National Cancer Intelligence Network data for 2012 [9], as it was both recent and nationally representative.

Collection of data of this type is improving in the UK, facilitated by initiatives such as the National Cancer Intelligence Network [20] and the England and Wales National Prostate Cancer Audit [21]. These data sets could be invaluable in refining our model in future years, and could yield extra information about the treatment choices made by urologists and others. Data such as these are important for assessing the extent to which men are receiving the best available treatments, and identifying areas where improvements could be made. In the absence of registry data for the time period of interest, we had to rely on data reported in the survey, recognising its limitations, to allow us to estimate the impact of the PR.3/PR07 and SPCG-7/SFUO-3 trials.

The results of trials are not the only factor that influences clinical decisions, so we cannot assume that a trial demonstrating a significant survival advantage from a treatment approach will automatically change practice. Research of this type is important to assess the extent to which knowledge is translated into changes in practice and, ultimately, patient benefits. This can help to demonstrate the value of clinical trials and identify where there is potential for further improvement in patient outcomes. Further research should be conducted to explore the impact of other practice-changing trials. This type of research is essential in demonstrating the value of clinical trials in advancing the outcomes of patients, regardless of the disease or intervention tested.

## 5 Conclusions

Clinicians in the UK and Canada have already responded to emerging evidence on the superiority of ADT + EBRT to ADT alone for treating locally advanced prostate cancer. The resulting changes in practice mean that collectively, men with this condition will survive longer. Further reductions in the numbers of men treated with ADT alone could lead to even better survival. ADT + EBRT is the only gold-standard therapeutic approach backed by level 1 evidence for the radical treatment of locally advanced prostate cancer. Until this changes, ADT + EBRT should be regarded as a standard of care for all men with locally advanced prostate cancer fit enough to receive radical local treatment.

  ***Author contributions:*** Annabelle South had full access to all the data in the study and takes responsibility for the integrity of the data and the accuracy of the data analysis.  

*Study concept and design:* South, Mason, Sydes, Parulekar.

*Acquisition of data*: South, Mason, Sydes, Parulekar.

*Analysis and interpretation of data:* South, Chen, Mason, Sydes, Parmar, Warde, Clarke.

*Drafting of the manuscript:* South, Mason, Sydes.

*Critical revision of the manuscript for important intellectual content:* South, Parulekar, Sydes, Chen, Parmar, Clarke, Warde, Mason.

*Statistical analysis:* South, Chen, Sydes.

*Obtaining funding:* None.

*Administrative, technical, or material support:* None.

*Supervision:* Sydes, Parmar, Mason.

*Other:* None.  

***Financial disclosures:*** Annabelle South certifies that all conflicts of interest, including specific financial interests and relationships and affiliations relevant to the subject matter or materials discussed in the manuscript (eg, employment/affiliation, grants or funding, consultancies, honoraria, stock ownership or options, expert testimony, royalties, or patents filed, received, or pending), are the following: Malcolm Mason has received fees for advisory board membership and lectures from Sanofi, Dendreon, Astellas, Johnson & Johnson, Ferring, Bayer, Caris, and Bristol-Myers Squibb. The remaining authors have nothing to disclose.  

***Funding/Support and role of the sponsor:*** The UK Medical Research Council funded time spent by A.S., M.R.S., and M.K.P. in working on this research. The sponsor played no role in the study.  

***Acknowledgments:*** The PR.3/PR07 trial was funded by the Canadian Cancer Society Research Institute (grant numbers 015469 and 021039), US National Cancer Institute (grant number CA077202), and the UK Medical Research Council. The authors would like to thank everyone who completed the survey and patients who participated in the PR.3/PR07 trial. The authors would also like to thank David Greenberg of the Eastern Office of the National Cancer Registration Service for providing data on the staging of prostate cancer diagnoses in the region and on treatment patterns for men with locally advanced prostate cancer.

## Figures and Tables

**Table:** 

1.	Which nation do you work in?
2.	What is your discipline?
	Clinical oncologist
	Medical oncologist
	Urologist
	Other
3.	What proportion of your patients with newly diagnosed locally advanced prostate cancer do you treat with the following approaches?
	Hormone therapy alone
	Radical prostatectomy
	External beam radiotherapy alone
	Hormone therapy and radiotherapy
	Other
4.	If you treat men with locally-advanced prostate cancer with an approach not listed in question 3, please specify what that approach is here
5.	Were you aware of the results of the MRC PR07 / NCIC PR.3 trial?
6.	Were you aware of the results of the SPCG-7/SFUO-3 trial?
7.	Have the results of the PR07/PR.3 and SPCG-7/SFUO-3 trials influenced how you treat patients with locally advanced prostate cancer?
8.	Do you think the evidence on adding radiotherapy to hormone therapy is strong enough for this to be the routine standard of care for men with locally advanced prostate cancer?
9.	How important are the following factors as barriers to hormone therapy + radiotherapy being the routine standard of care for men with locally advanced prostate cancer?
	Availability / waiting times locally
	Attitudes of patients
	Attitudes of urology colleagues
	Attitude of oncology colleagues
	Attitude of nursing colleagues
	Toxicity concerns
	Evidence base
	Other approaches are better
10.	Are you currently supporting any trials that recruit patients with locally advanced prostate cancer?
11.	What is the most pressing research question for men with locally advanced prostate cancer currently?
12.	Any other comments?

**Table 1:** Speciality of survey respondents by country

	Survey respondents, *n* (%)			
	Canada	UK	Other	Total
Clinical/radiation oncologist	58 (73)	71 (68)	6 (75)	135 (70)
Medical oncologist	10 (13)	6 (6)	0 (0)	16 (8)
Urologist	8 (10)	19 (18)	2 (25)	29 (15)
Other	4 (5)	9 (9)	0 (0)	13 (7)
Total	80 (100)	105 (100)	8 (100)	193 (100)

**Table 2:** Current approaches for treatment of locally advanced prostate cancer by country

Therapeutic approach	Canada, *n* (%)	UK, *n* (%)	*p* value (Canada vs UK)	Other, *n* (%)	Total, *n* (%)
ADT alone			0.002		
None	9 (13)	1 (1)		3 (38)	13 (8)
<10%	51 (71)	53 (60)		3 (38)	107 (64)
10–50%	10 (14)	31 (36)		0	41 (25)
51–90%	2 (3)	2 (2)		2 (25)	6 (4)
>90%	0	1 (1)		0	1 (1)
EBRT alone			0.001		
None	18 (31)	47 (65)		4 (50)	69 (50)
<10%	25 (43)	17 (24)		1 (13)	43 (31)
10–50%	12 (21)	7 (10)		2 (25)	21 (15)
51–90%	3 (5)	1 (1)		1 (13)	5 (4)
>90%	0	0		0	0
ADT + EBRT			0.041		
None	2 (3)	0		0	2 (1)
<10%	0	0		1 (13)	1 (1)
10–50%	15 (20)	18 (18)		3 (38)	36 (20)
51–90%	30 (39)	57 (58)		2 (25)	89 (49)
>90%	29 (38)	24 (24)		2 (25)	55 (30)
Radical prostatectomy			0.012		
None	27 (44)	19 (23)		2 (25)	48 (32)
<10%	21 (34)	44 (53)		4 (50)	69 (45)
10–50%	11 (18)	20 (24)		2 (25)	33 (22)
51–90%	2 (3)	0		0	2 (1)
>90%	0	0		0	0
Other treatment			0.319		
None	24 (67)	19 (46)		3 (60)	46 (56)
<10%	9 (25)	16 (38)		2 (40)	27 (33)
10–50%	2 (6)	5 (12)		0	7 (9)
51–90%	1 (3)	1 (2)		0	2 (2)
>90%	0	0		0	0

ADT = androgen deprivation therapy; EBRT = external beam radiation.

**Table 3:** Life years saved in a cohort of 7930 men diagnosed in 1 yr by moving from androgen deprivation therapy (ADT) alone to ADT with external beam radiation (EBRT)

Scenario	Treatment (%)		Men treated with ADT alone per year (*n*)	Change in men treated with ADT alone vs reference (*n*)	Life-years in cohort saved	
	ADT + EBRT	ADT alone				
					10 yr	15 yr
Reference	17	73	5789	0	0	0
1	52	24	1903	–3886	2021	3730
2	62	19	1507	–4282	2227	4111
3	72	16	1269	–4520	2350	4339
4	82	10	793	–4996	2598	4796
5	92	5	396	–5392	2804	5177

**Table 4:** Awareness of and views on evidence about androgen deprivation therapy (ADT) with external beam radiotherapy (EBRT) by country

	Survey respondents, *n* (%)			
	Canada	UK	Other	Total
Awareness of trial results				
Aware of PR.3/PR07 results?				
Yes, read *Lancet* paper	60 (81)	82 (80)	8 (100)	150 (81)
Yes, other source	12 (16)	19 (18)	0	31 (17)
No	2 (3)	2 (2)	0	4 (2)
Aware of SPCG7/SFUO3 results				
Yes, read *Lancet* paper	42 (58)	59 (58)	6 (86)	107 (59)
Yes, other source	13 (18)	21 (21)	1 (14)	35 (19)
No	18 (25)	22 (22)	0	40 (22)
Have the results of these trials influenced your clinical practice?				
Already generally using ADT + EBRT	58 (77)	56 (56)	6 (75)	120 (66)
Now generally use ADT + EBRT	16 (21)	44 (44)	2 (25)	62 (34)
Not generally using ADT + EBRT	1 (1)	0	0	1 (1)
Is the evidence strong enough for ADT + EBRT to be the standard of care for men with locally advanced prostate cancer?				
Yes	68 (91)	89 (88)	6 (75)	163 (89)
No	4 (5)	4 (4)	1 (13)	9 (5)
Not sure	3 (4)	8 (8)	1 (13)	12 (7)

**Table 5:** Barriers to the use of androgen deprivation therapy with external beam radiotherapy as the routine standard of care for men with locally advanced prostate cancer by country

	Survey respondents, *n* (%)			
	Canada	UK	Other	Total
Availability/waiting times locally				
Not a barrier	66 (86)	84 (82)	8 (100)	158 (84)
Slight barrier	8 (10)	14 (14)	0	22 (12)
Moderate barrier	3 (4)	2 (2)	0	5 (3)
Major barrier	0	3 (3)	0	3 (2)
Attitudes of patients				
Not a barrier	32 (42)	63 (62)	3 (38)	98 (52)
Slight barrier	36 (47)	32 (31)	4 (50)	72 (39)
Moderate barrier	9 (12)	7 (7)	1 (13)	17 (9)
Major barrier	0	0	0	0
Attitudes of urology colleagues				
Not a barrier	31 (41)	64 (63)	1 (13)	96 (52)
Slight barrier	28 (37)	27 (27)	2 (25)	57 (31)
Moderate barrier	15 (20)	8 (8)	4 (50)	27 (15)
Major barrier	1 (1)	2 (2)	1 (13)	4 (2)
Attitude of oncology colleagues				
Not a barrier	62 (82)	87 (86)	7 (88)	156 (84)
Slight barrier	12 (16)	12 (12)	1 (13)	25 (14)
Moderate barrier	2 (3)	2 (2)	0	4 (2)
Major barrier	0	0	0	0
Attitude of nursing colleagues				
Not a barrier	74 (97)	90 (89)	7 (88)	171 (92)
Slight barrier	2 (3)	11 (11)	1 (13)	14 (8)
Moderate barrier	0	0	0	0
Major barrier	0	0	0	0
Toxicity concerns				
Not a barrier	13 (18)	34 (34)	0	47 (26)
Slight barrier	43 (58)	54 (53)	3 (38)	100 (55)
Moderate barrier	18 (24)	12 (12)	4 (50)	34 (19)
Major barrier	0	1 (1)	1 (13)	2 (1)
Evidence base				
Not a barrier	62 (82)	76 (75)	6 (75)	144 (78)
Slight barrier	9 (12)	22 (22)	1 (13)	32 (17)
Moderate barrier	3 (4)	3 (3)	0	6 (3)
Major barrier	2 (3)	0	1 (13)	3 (2)
Other approaches are better				
Not a barrier	62 (91)	73 (85)	6 (75)	141 (87)
Slight barrier	4 (6)	13 (15)	2 (25)	19 (12)
Moderate barrier	2 (3)	0	0	2 (1)
Major barrier	0	0	0	0

## Appendix A

Survey questions
